# Supplementary material for: Evidence supported by Mendelian randomization: impact on inflammatory factors in knee osteoarthritis
Source: Front Med (Lausanne). 2024 May 28;11:1382836. doi: 10.3389/fmed.2024.1382836 (PMC11165061; doi:10.3389/fmed.2024.1382836)
Supplement: Supplementary file 1 [file Data_Sheet_1.ZIP › data/GCST90274774_5e6/774.leaveoneout.pdf]

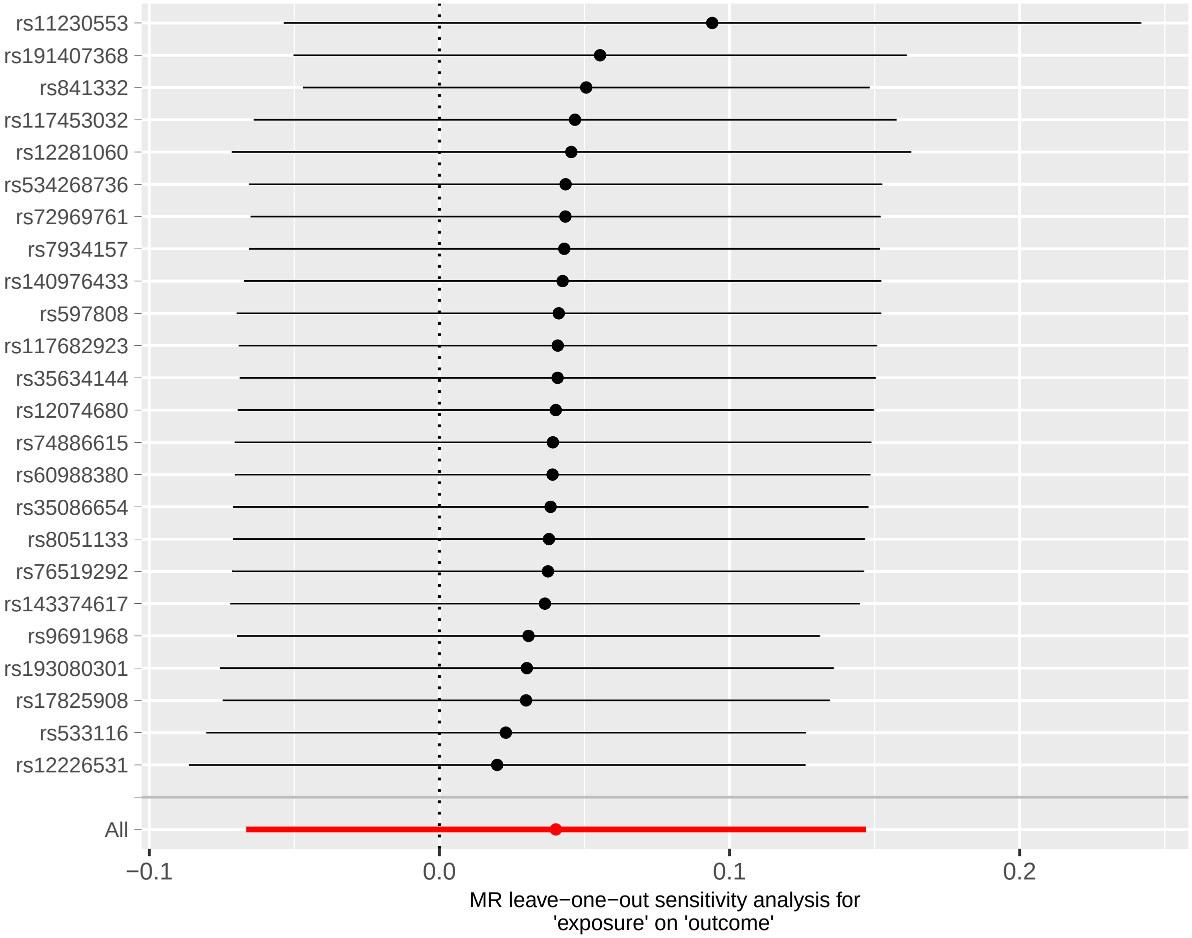
rs11230553
rs191407368
rs841332
rs117453032
rs12281060
rs534268736
rs72969761
rs7934157
rs140976433
rs597808
rs117682923
rs35634144
rs12074680
rs74886615
rs60988380
rs35086654
rs8051133
rs76519292
rs143374617
rs9691968
rs193080301
rs17825908
rs533116
rs12226531
All
−0.1
0.0
0.1
0.2
MR leave−one−out sensitivity analysis for
'exposure' on 'outcome'
